# Enhanced Delivery of Lipid Nanoparticle-Based Immunotherapy by Modulating the Tumor Tissue Stiffness Using Ultrasound-Activated Nanobubbles

**DOI:** 10.1021/acsnano.5c21787

**Published:** 2026-01-28

**Authors:** Anubhuti Bhalotia, Diarmuid W. Hutchinson, Theresa Kosmides, Pinunta Nittayacharn, Meghna Mehta, Arya Iyer, Andrew Cheplyansky, Koki H. Takizawa, Abraham Nidhiry, Anna M. Dever, Kyle A. Cousens, Inga M. Hwang, Gopalakrishnan Ramamurthy, Agata A. Exner, Efstathios Karathanasis

**Affiliations:** † Department of Biomedical Engineering, School of Medicine, 2546 Case Western Reserve University, Cleveland, Ohio 44106, United States; ‡ Department of Biomedical Engineering, Faculty of Engineering, 68000 Mahidol University, Phutthamonthon, Nakhon Pathom 73170, Thailand; § Department of Radiology, School of Medicine, Case Western Reserve University, Cleveland, Ohio 44106, United States; ∥ Case Comprehensive Cancer Center, School of Medicine, Case Western Reserve University, Cleveland, Ohio 44106, United States

**Keywords:** ultrasound, nanobubbles, tumor tissue stiffness, lipid nanoparticles, gene delivery, immunotherapy

## Abstract

Tumors often exhibit an extracellular matrix with elevated stiffness due to excessive accumulation and cross-linking of proteins, particularly collagen. This elevated stiffness acts as a physical barrier, impeding the infiltration of immune cells and the effective delivery of various immunotherapeutic agents, such as lipid nanoparticle-based RNA therapeutics. Here, we investigate the ability of ultrasound-activated nanobubbles (US-NBs) to increase the permeability and immunogenicity of tumors. Our results show that US-NBs physically remodel the tumor tissue by decreasing its stiffness by 60% 5 days after a single treatment. US-NB-treated tumors display randomly oriented collagen with a 5.47-fold lower deposition compared to untreated tumors. This leads to the effective delivery and widespread distribution of lipid nanoparticles (LNPs) in the tumor. Importantly, when assisted by US-NB, LNPs exhibit superior gene-transfection efficiency across pan-immune cells and achieve efficient genetic modification of T cells directly in vivo. This combined approach engages both innate and adaptive immunity, enhancing tumor immunogenicity and boosting cytotoxic cell infiltration by 4-fold compared to LNPs alone. These results indicate that gentle mechanical stimulation of the tumor using US-NB offers a promising strategy to augment the delivery and efficacy of existing immunotherapies.

## Graphical abstract

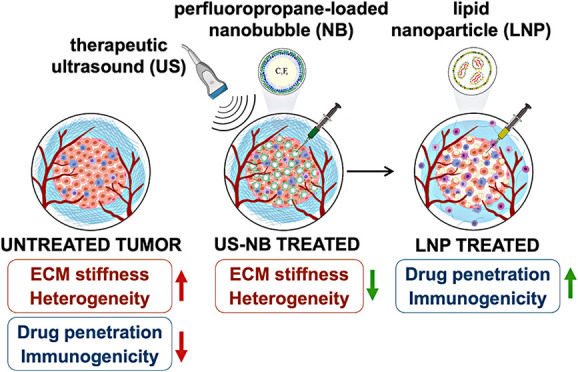

Cancer immunotherapies often fail because cytotoxic T lymphocytes are marginalized and excluded from cancer masses,^1–3^ due to significant challenges, including massive immunosuppression^2^ and elevated extracellular matrix (ECM) stiffness.^5–7^ The interplay between the tumor microenvironment and the ECM stiffness is established as a critical factor in regulating the malignancy and immunogenicity of growing tumors.^8^ Tumors remodel their ECMs, which are manifested by an excessive accumulation and cross-linking of ECM proteins, particularly collagen.^9^ First, the stiff tumor ECM compromises the proper function of both innate and adaptive immune cells.^10–12^ Beyond this biological suppression, the elevated stiffness acts as a physical barrier that impedes the infiltration of immune cells and the effective delivery of a broad spectrum of immunotherapeutic agents, including monoclonal antibodies, adoptive cell therapies, and vaccines.^13–15^ Crucially, this barrier restricts distribution not only from the vasculature but even following direct intratumoral administration.^16,17^

Ultrasound-activated (US) nanobubbles (NBs) offer a mechanical solution to the restricted interstitial transport.^18^ NBs are echogenic perfluoropropane gas bubbles with compressible gas cores and robust yet deformable phospholipid shells.^19–21^ Their resilient shells and nanoscale diameters allow them to penetrate deep into tissue, which would otherwise exclude microbubbles.^22–26^ Due to these properties, NBs efficiently distribute throughout the entire tumor mass, creating widespread reservoirs of tiny gas bubbles. Stimulation of the NBs with ultrasound induces stable or inertial bubble cavitation, which, in turn, generates microstreaming and microjets, resulting in mechanical (or thermal) effects on the surrounding tissues.^27^ Our hypothesis is that gentle mechanical stresses under mild therapeutic ultrasound generated by US-NB leads to restoration of the tumor ECM elasticity without damaging the tissue.^28,29^ Here, we present the US-NB therapeutic strategy that mechanically modifies the tumor microenvironment to overcome these physical barriers, priming the tumor for improved delivery of existing immunotherapies and efficient infiltration of immune cells.

Several studies have explored the application of ultrasound to augment the efficacy of immunotherapy.^30–34^ These prior efforts leveraged microbubble cavitation to enhance delivery of monoclonal antibodies (mAbs) for immune checkpoint blockade or immunomodulatory agents (e.g., toll-like receptor agonists). These advances have established the principle that mechanical forces can modulate the tumor microenvironment. However, the rapidly emerging class of RNA-based lipid nanoparticles (LNPs) presents a distinct therapeutic opportunity^35,36^ that remains largely unexplored in the context of ultrasound-mediated delivery. LNPs are constrained by the dense tumor tissue,^37,38^ and their nanoscale dimensions may require complementary delivery strategies beyond those optimized for smaller molecular therapeutics. Our approach focused on using ultrasound-activated nanobubbles to physically remodel the tumor microenvironment, reducing stiffness and enabling improved delivery and infiltration of LNPs throughout the tumor tissue.

In this work, using a murine model of breast cancer, we assess the impact of US-NB on the tumor ECM and subsequent drug delivery. Using ultrasound imaging, we show that intratumorally administered NBs efficiently distribute throughout the entire tumor. The widespread distribution of NBs in the tumor produces a remarkable decrease of the tumor ECM stiffness after the application of therapeutic ultrasound as measured with ultrasound elastography and histological analysis. To determine whether this ECM remodeling translates to improved therapeutic outcomes, we chose an LNP-based immunotherapy with a standard LNP size, which has been shown to constrain their penetration and distribution within tumors. The LNP was designed for immune checkpoint blockade therapy using local intratumoral delivery and gene silencing. As cargo of the LNPs, we selected siRNAs targeting PD-1 and CTLA-4,^39^ which is a clinically validated combination of immune checkpoint inhibitors (using mAbs). This approach allowed us to assess whether US-NB simultaneously affects both innate and adaptive immune compartments. Even with local administration, LNPs alone could not bypass the physical tumor barriers and disperse effectively throughout the tumor tissue. However, when used in conjunction, US-NB enabled the uniform dispersion of LNPs in the tumor and increased the delivery and concentration of LNPs to immune cells, remarkably even T cells. As a result of US-NB, LNPs efficiently delivered their gene cargo directly to T cells in vivo, which are typically inaccessible to delivery systems.^40–43^ US-NB-enhanced LNP delivery simultaneously improved antigen-presenting cell expansion and activated both CD4^+^ and CD8^+^ T cell populations throughout the tumor, showing that mechanical remodeling of the microenvironment is an effective strategy to enhance immunotherapy efficacy.

## Results

### Nanobubbles Achieve Widespread Distribution throughout an Entire Tumor

The compressible gas core, deformable shell, and nanoscale size allow NBs to rapidly spread throughout dense tumor tissues. We characterized NBs filled with C_3_F_8_ using dynamic light scattering, resonant mass measurement, and ζ-potential analysis. NBs exhibited a hydrodynamic diameter of 283 nm with excellent agreement between intensity and number-weighted size distributions (Figure 1a). Due to their resilient shell, nearly 90% of the particles were acoustically buoyant (Figure 1b). The NBs also possessed a slightly negative surface charge of −34 mV in PBS at pH 7.4, which discouraged aggregation (Figure 1c). Together, these properties (nanoscale dimensions, acoustic responsiveness, deformability, and colloidal stability) enabled rapid and uniform distribution throughout the tumor tissue upon injection.

**Figure 1:**
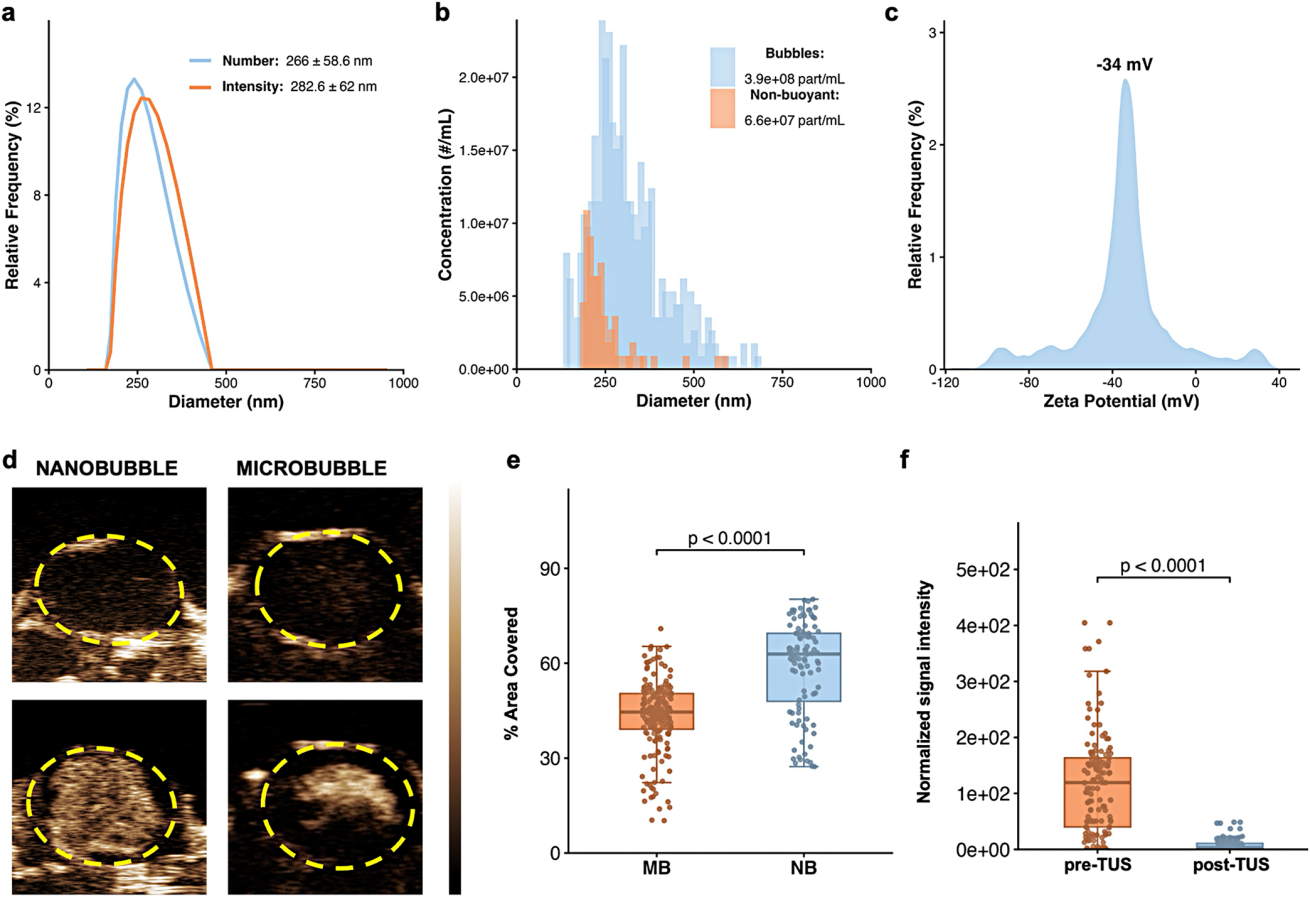
Ultrasound-activated nanobubble characterization and in vivo tumor perfusion. (a) Hydrodynamic diameter of nanobubbles in PBS, reported as both intensity- and number-weighted distributions. (b) Resonant Mass Measurement (RMM) characterizing nanobubble acoustic buoyancy and concentration. (c) ζ-potential of nanobubbles measured in PBS at room temperature. (d) Representative static frames from Contrast-Enhanced Ultrasound (CEUS) imaging at the center of an E0771.LMB tumor. Images show the tumor at baseline, following nanobubble (NB) or microbubble (MB) injection. (e) Quantification of the percent contrast-covered area per tumor region of interest (ROI) derived from volumetric CEUS scanning; comparison is made between nanobubbles and microbubbles. (f) Quantification of the normalized contrast signal intensity using the raw, linear data across tumor volumetric frames; comparison is made for nanobubbles pre- and post-TUS application. Statistics were performed via Wilcox on matched-pairs signed rank test (*n* = 3 mice per group, data points represent frames taken across the tumor length for each animal).

Nanobubbles were originally developed to overcome the limitations of microbubbles in systemic delivery, where their smaller size improved extravasation from the vasculature into tissue. We wanted to test whether this advantage translated to the intratumoral platform, where a different barrier exists of penetration through the dense tumor periphery following direct tissue injection. We compared NBs to MBs formulated with identical lipid shells but sized at 690 nm (Figure S1), representative of clinically used formulations. When administered intratumorally, microbubbles remained largely confined to the injection site, with limited penetration into the dense periphery. NBs, by contrast, penetrated uniformly from the tumor center outward to the periphery immediately upon administration (Figure 1d). Quantitative analysis of volumetric CEUS scans revealed that NBs covered approximately 60% of each tumor slice, compared to 40% for microbubbles (Figure 1e). While both formulations filled the central injection region, only NBs achieved substantial filling throughout the periphery.

To maximize the cavitational impact of NBs on the ECM, therapeutic ultrasound (3.3 MHz, 2.2 W, 50% duty cycle, 1 min) was applied immediately following injection, at a point when NBs were highly stable and distributed throughout the tumor. Previous studies from our group^44^ optimized the ultrasound parameters for therapeutic applications of NBs injected intravenously. For intratumoral translation, the treatment duration was deliberately reduced to limit mechanical agitation and achieve gentle disruption of the extracellular matrix (ECM). In turn, the duty cycle was increased to 50% to enable efficient cavitation of NBs distributed through densely packed tissue. To assess any thermal interference from cavitation, surface temperatures were measured using an IR thermometer, showing no elevation from the initial precavitation baseline temperature (36.8 °C). Immediately post-TUS, CEUS imaging showed efficient bubble cavitation, with quantitative analysis revealing a ∼11-fold loss of signal intensity (Figure 1f), indicating near-complete acoustic destruction of the NB population. This efficient cavitation generates mechanical forces (jet streams and shock waves) that can disrupt the extracellular matrix leading to improved tissue permeability.

The timing of ultrasound application was deliberate. NBs have maximum internal gas pressure immediately after generation, providing optimal conditions for cavitation. We selected tumors in the 40 to 60 mm^3^ size range to ensure the tumor microenvironment was fully established, with mature ECM density, developed vasculature, and defined immune composition, yet still in exponential growth with minimal necrotic core. This narrow size window provided a consistent baseline for TME features across experiments. By applying TUS immediately after NB injection, once complete tumor filling had occurred but before significant cellular internalization, we ensured that the cavitational energy was directed primarily at ECM remodeling. The resulting mechanical stresses (jet streams, shock waves, and acoustic radiation forces) preferentially disrupted the extracellular matrix, increasing its permeability without excessive cellular damage. This precision in both timing and mechanical targeting positions ultrasound-activated NBs as an effective strategy for ECM remodeling and improved therapeutic delivery in solid tumors.

### Nanobubble Cavitation Reduces the ECM Stiffness of the Tumor

Cavitation of NBs generates mechanical forces including jet streams and shock waves. By cavitating the NBs when they possessed both high internal pressure and complete tumor distribution, we maximized their cavitational impact on the ECM. This mechanical disruption can increase the permeability of the matrix, which subsequently should facilitate deeper penetration of drugs. To assess the mechanical changes, ultrasound shear wave elastography (SWE) was used to quantify tumor stiffness and qualitatively assess tissue heterogeneity. The noninvasive nature of SWE allows for serial in vivo measurements within the same tumor, allowing us to track ECM remodeling over time following a single US-NB application.

We first validated SWE measurements using polyacrylamide phantoms (2 and 40 kPa), which returned accurate elastic moduli with high signal-to-noise ratios and homogeneous elastograms (Figure S2). In vivo elastography was performed at the tumor center with a standoff gel pad maintaining reproducible focal depth. ROI grids were defined to encompass the entire tumor slice (Figure 2a), as shown in representative B mode images. The uncropped elastograms are shown in Figure S3a. Elastic moduli were determined by averaging triplicate measurements at distinct sampling points within each tumor. Following treatment, SWE measurements were taken immediately postultrasound and then daily through day 5. Measurements were concluded when tumor growth exceeded ROI grid dimensions, ensuring consistent measurement conditions. Daily caliper measurements confirmed that tumor volume remained relatively stable throughout the measurement period (Figure S3b), allowing us to isolate the effects of ECM remodeling independent of tumor regression.

**Figure 2:**
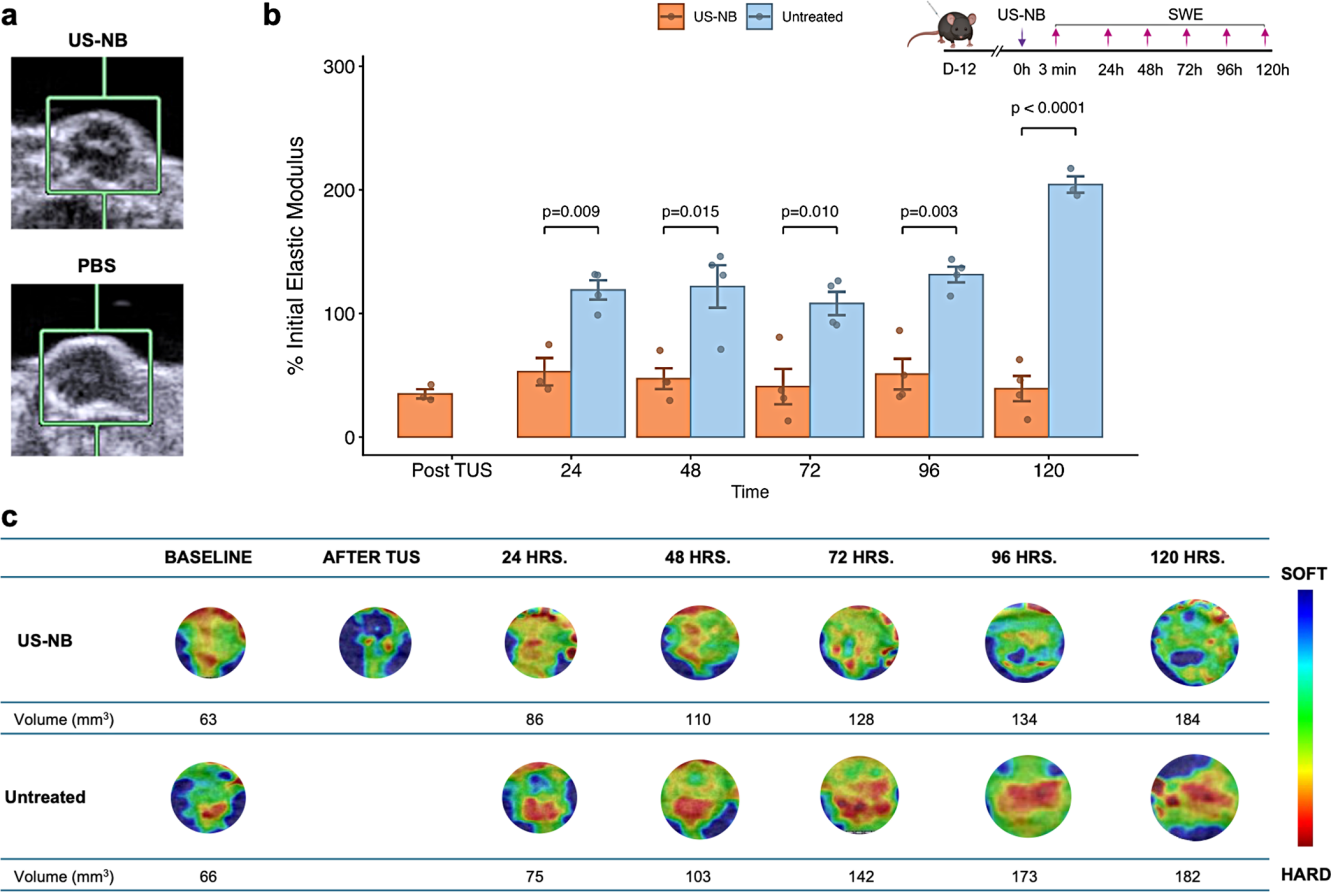
Evaluation of tumor stiffness with Ultrasound Shear Wave Elastography (SWE). (a) Representative B-mode images of E0771.LMB tumors used for quantitative measurements of the elastic modulus. (b) Quantitative measurements of elastic moduli over time, every day for 5 days (*n* = 4 mice per group). Stiffness normalized to the initial values of the tumor. Only the treated group is evaluated immediately post-TUS treatment, assuming no change in the untreated group, 3 min post injection. Statistics were conducted using multiple unpaired *t* test with a two-stage step-up post hoc test at 1% false discovery rate. Resulting *p* values are reported. (c) Qualitative elastograms are cropped to the tumor ROI and reported with the range of soft to hard tissue. Corresponding tumor volumes obtained through calipering are reported. ROIs are kept to scale.

Quantitative measurements revealed substantial changes in tumor stiffness upon treatment (*q* = 0.0001). As expected, the untreated (PBS) controls progressively stiffened to a final ∼204% of their initial elastic modulus by day 5 (Figure 2b). Comparatively, the elastic modulus of the tumor decreased to 35% of its pretreatment value immediately after US-NB. Remarkably, US-NB-treated tumors sustained this lowered stiffness (39% of the initial) for the duration of SWE measurements. The sustained 3-fold reduction in tumor stiffness indicates successful physical remodeling of the ECM which has been correlated to a more permeable tissue structure.

Beyond reducing bulk stiffness, US-NB treatment also reduced the spatial heterogeneity of tissue stiffness. Elastograms displaying topological stiffness maps revealed significant differences between treated and untreated tumors (Figure 2c, with original images in Figure S2). Untreated (PBS-injected) tumors developed increasingly heterogeneous stiffness distributions over time, with rigid regions progressively expanding from the tumor core toward the periphery. Following a single US-NB treatment, tumors progressively reduced their heterogeneity, indicating successful physical dismantling of the ECM architecture. This reduction in spatial heterogeneity is significant, as tumor stiffness heterogeneity supports treatment-resistant growth and metastatic potential, while normalized tissue architecture promotes improved infiltration of both therapeutics and immune cells.

The immediate and sustained reduction in both ECM stiffness and tissue heterogeneity demonstrates successful physical remodeling by ultrasound-activated NB cavitation. Stiff and heterogeneous ECMs are hallmarks of aggressive and metastatic cancers. Our results show that US-NB treatment reverts tumor ECM toward a more normalized structure, creating conditions permissive for uniform drug and immune cell distribution throughout the tumor.

### Nanobubble Cavitation Remodels the Extracellular Matrix

US-NB treatment sustained reductions in tumor stiffness and heterogeneity, indicating successful ECM remodeling. To understand the cellular and structural mechanisms underlying these mechanical changes, we assessed cell viability, tissue organization, and collagen content in E0771.LMB tumors at volumes of 40–60 mm^3^ and harvested 24 h post-treatment. This characterization was essential for establishing safety in the context of immunotherapy, where preserving functional immune cells is critical for therapeutic efficacy.

US-NB treatment reduced cellular density while preserving cell viability. DAPI staining revealed that only the US-NB group exhibited significant reduction in cell density compared to both ultrasound alone (US, *p* = 0.0157) and untreated controls (PBS, *p* = 0.002) (Figure 3a). This decrease reflected physical displacement of cells during cavitation rather than cell death, as cleaved caspase 3 staining showed no increase in apoptosis in US-NB treated tumors, with mean fluorescence intensity indistinguishable from controls (Figure 3b). US controls showed no difference from untreated tumors, confirming that mechanical disruption required cavitation-induced forces, not ultrasound alone.

**Figure 3:**
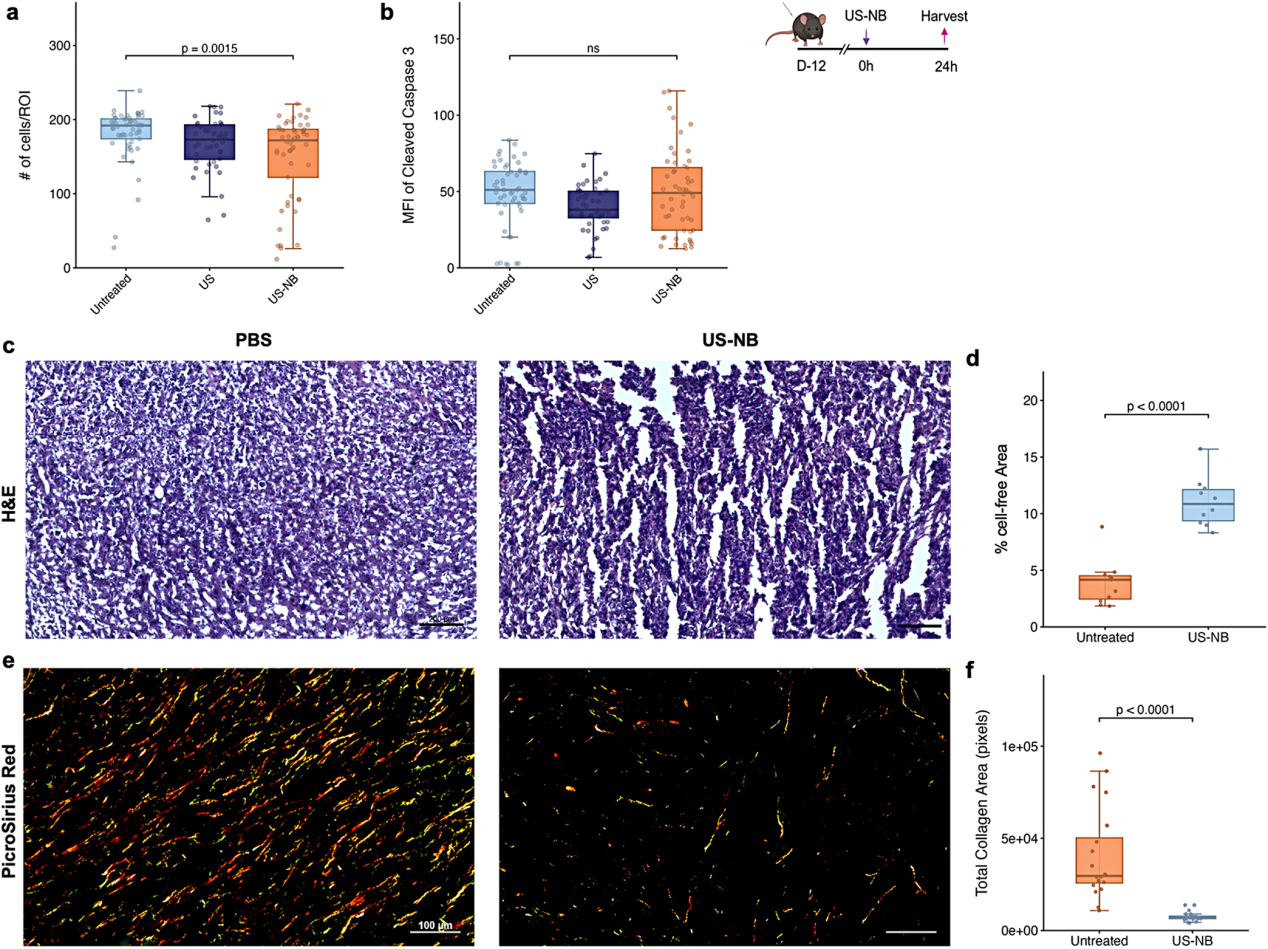
Evaluation of extracellular remodeling and apoptosis in the tumor microenvironment. E0771.LMB tumors were treated at 40–60 mm^3^ and harvested 24 h post-treatment for histological analysis (*n* = 5 mice per group). Immunohistochemistry was performed using staining for Cleaved Caspase-3 (an apoptosis marker) and DAPI as a nuclear counterstain. Quantification of (a) DAPI^+^ cell counts per region of interest (ROI), and (b) Cleaved Caspase-3 mean fluorescence intensity (MFI). Comparisons were made between Untreated, US (Ultrasound only), and US-NB (Ultrasound + Nanobubbles) groups using a one-way ANOVA followed by Tukey’s post hoc test comparing the mean of each group to the others. (c) Representative H&E staining showing tissue integrity. Scale bars represent 200 μm. (d) Quantification of acellular area per ROI as a measure of structural disruption. (e) Representative Picrosirius Red staining visualized under polarized light to assess collagen content. Scale bars represent 100 μm. (f) Quantification of total collagen area per ROI. ROIs were of the same size and resolution across all analyzed images. For (d,f), comparisons were conducted between Untreated and US-NB groups using a Student’s *t* test with Welch’s correction.

US-NB altered the spatial arrangement of cells within the tissue by disrupting structural cohesion. H&E staining 24 h post-treatment revealed substantial changes in tissue architecture (Figure 3c). US-NB generated a 3.53-fold increase in acellular regions compared to PBS controls (Figure 3d), indicating that cavitation-induced mechanical forces had physically reorganized the tissue landscape. Notably, no necrotic regions were observed, confirming that tissue remodeling occurred without triggering cell death.

The mechanism driving this tissue disruption was collagen degradation. Picrosirius Red staining showed that untreated tumors contained dense, highly aligned collagen characteristic of breast cancer (Figure 3e). In contrast, US-NB-treated tumors exhibited fragmented collagen with a 5.47-fold reduction in total collagen area (Figure 3f). Additional representative histological images comparing the center and periphery of the tumors are shown in Figure S4. Beyond the magnitude of collagen reduction, US-NB disrupted collagen organization itself, fragmenting the fibrous network that normally constrains drug diffusion. These structural changes directly reduce physical barriers to therapeutic penetration and enable more uniform drug distribution throughout the tumor.

Solid tumors leverage a dense ECM to support growth and establish a challenging barrier to drug delivery. By mechanically dismantling tissue architecture and degrading the collagen network, US-NB increases tumor permeability while maintaining cellular viability. This combination of improved penetration and preserved cell function creates conditions favorable for immunotherapeutic efficacy, enabling therapeutic agents to reach immune cells distributed throughout the tumor without compromising their function.

### Nanobubble Cavitation Can Directly Deliver Nanoparticles to T Cells and Tumoral Periphery

To assess whether US-NB could mechanically transform the tumor microenvironment for better therapeutic efficacy, we investigated immune checkpoint-silencing LNPs. The LNPs, with diameters of 50–80 nm (Figure S5a), neutral surface charge (Figure S5b), and >90% siRNA encapsulation (Figure S5c), represent an optimal design for local delivery. Nevertheless, these nanoparticles continue to encounter major physical barriers within solid tumors—including a rigid ECM, poor penetration beyond the injection site, and insufficient access to T cells.

US-NB enhanced LNP spatial distribution throughout the entire tumor. Fluorescent LNPs were injected at the tumor center, and tumors were harvested 24 h post-treatment for histological analysis. Without US-NB, LNPs remained confined to the injection core (Figure 4a, left panel). With US-NB treatment, LNPs dispersed uniformly throughout the tumor, including substantial accumulation at the periphery (Figure 4a, right panel). Quantitatively, the tumor area containing LNPs increased by 20% (Figure 4b), and the periphery-to-center LNP ratio (Figure 4c) increased by ∼4-fold, indicating that US-NB treatment normalized distribution across the entire tumor volume.

**Figure 4:**
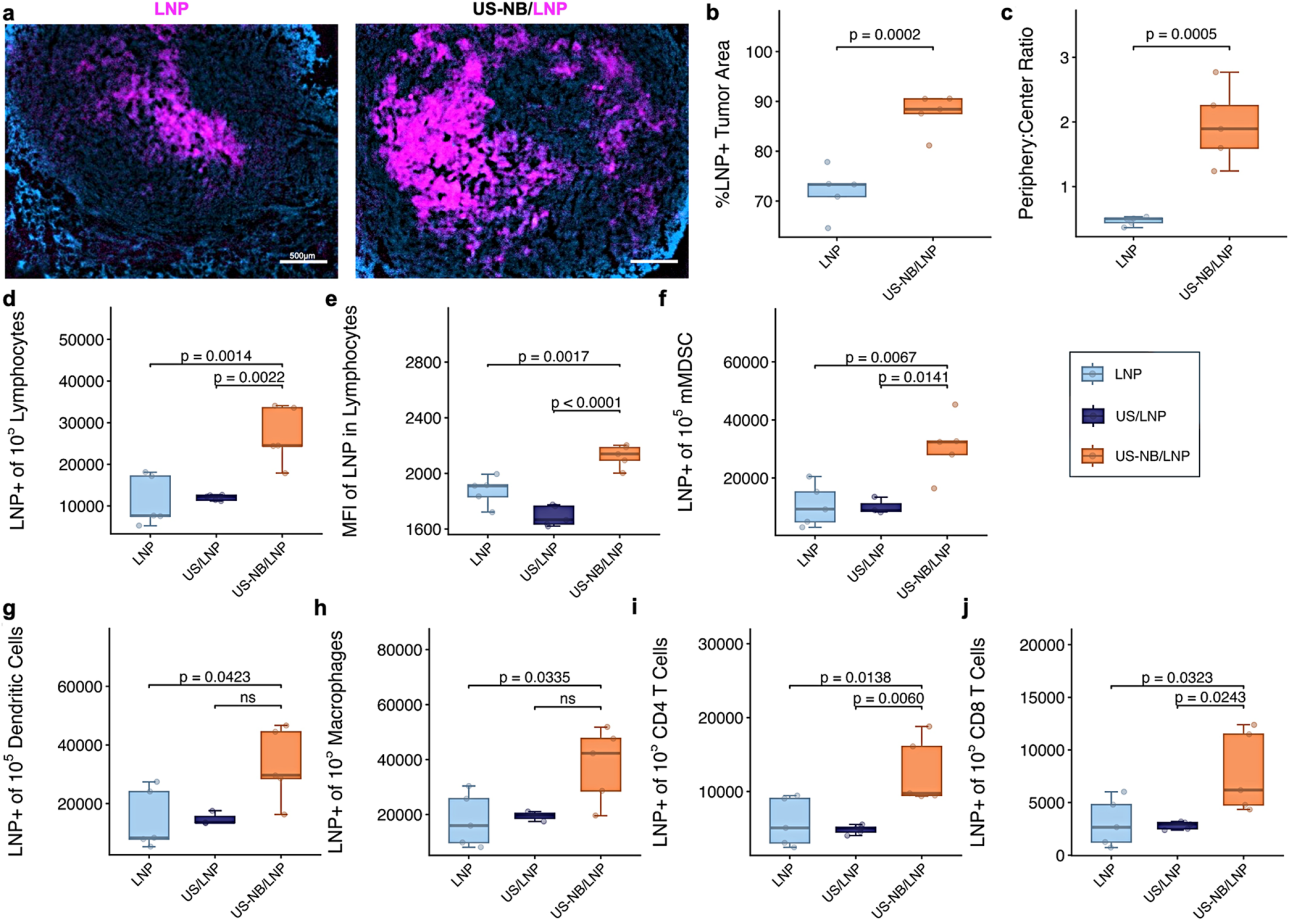
Spatial distribution and cellular uptake of LNPs in vivo. Fluorescent LNPs were administered intratumorally into E0771.LMB tumors. (a–c) Histological analysis of tumors harvested 24 h post-treatment (*n* = 3). (a) Representative fluorescence microscopy of DiR^+^ LNP distribution (Zeiss Axio Z1, 5×). (b) LNP tumor coverage calculated as the percentage of LNP^+^ area normalized to DAPI^+^ area. (c) Ratio of LNP fluorescence in the tumor periphery versus center (MATLAB segmentation). (d–j) Flow cytometry assessment of cellular uptake 3 h postinjection in tumors treated with US-NB/LNP, US/LNP, or LNP (*n* = 5). (d) Frequency of LNP^+^ cells within the CD45^+^ immune population and (e) corresponding mean fluorescence intensity (MFI). Uptake was similarly quantified in (f) mMDSCs, (g) dendritic cells, (h) macrophages, (i) CD4^+^ T cells, and (j) CD8^+^ T cells. Injection volumes were constant. Statistical significance was determined using an unpaired Student’s *t* test with Welch’s correction (b, c) or a one-way ANOVA followed by Tukey’s post hoc test (d–j).

US-NB increased both the number of immune cells internalizing LNPs and the concentration of LNPs per cell. E0771.LMB tumors were harvested 3 h after treatment with US-NB/LNP, US/LNP, or LNPs alone. Flow cytometry was used to assess the cell uptake of LNPs. The flow gating strategy is shown in Figure S6. US-NB/LNPs were internalized by 2.3-fold more immune cells compared to controls (Figure 4d). Additionally, the number of LNPs accumulated per immune cell were almost doubled for US-NB/LNPs (Figure 4e), indicating not just broader cellular targeting but also increased payload delivery to each cell.

US-NB markedly enhanced LNP delivery to both innate and adaptive immune cells critical for antigen-mediated responses. Delivery to innate effectors increased substantially: mMDSCs showed approximately 2-fold higher LNP uptake (Figure 4f), while dendritic cells and macrophages demonstrated 3-fold and 2.5-fold increases, respectively (Figure 4g,h). More significantly, T cell targeting improved dramatically. Because T cells typically resist nanoparticle internalization due to their limited endocytic capacity, LNP delivery to T cells is particularly challenging. US-NB/LNPs overcame this limitation, resulting in LNPs being internalized by approximately 3-fold more CD4^+^ T cells (Figure 4i) and approximately 3-fold more CD8^+^ T cells (Figure 4j) compared to LNPs alone.

Together, these results demonstrate that US-NB enables nanoparticle-based therapies to overcome key delivery barriers in solid tumors. By distributing LNPs broadly throughout the tumor and increasing their internalization across multiple immune cell populations, particularly T cells, US-NB substantially improves the likelihood that immuno-therapeutics will reach and engage their target immune cells. This represents a meaningful advancement in addressing the physical barriers that have limited immunotherapy efficacy in solid tumors.

### Nanobubble Cavitation Enhances Genetic Transfection

Since US-NB/LNPs had higher cellular accumulation, we next investigated whether this approach could also enhance LNP-mediated gene transfection which is frequently limited by inefficient endosomal escape even after successful cellular uptake. To focus specifically on transfection efficiency without confounding biological effects, we used an exogenous reporter (eGFP mRNA) in LNPs formulated by syringe mixing, yielding particles with a hydrodynamic diameter of ∼69 nm.

US-NB augmented LNP-mediated transfection and gene expression levels in pan-immune cells (Figure 5). Tumor volumes were compared to an untreated group, to confirm no changes in growth rates. E0771.LMB tumors were treated with eGFP mRNA loaded LNPs and harvested 24 h later. Flow cytometry was used to quantify GFP expression specifically among LNP-positive cells in each immune population. US-NB increased the transfection efficacy of LNPs across pan-immune (CD45^+^) cells by 1.4-fold (Figure 5a). Moreover, US-NB resulted in higher MFI (Figure 5b), indicating an increase in the GFP expression. Notably, activated macrophages, dendritic cells, and mMDSCs all showed ∼1.5-fold increases in transfection with US-NB (Figure 5c–e). Overall, US-NB caused an increase in the number of cells being transfected as well as the levels of gene expression.

**Figure 5:**
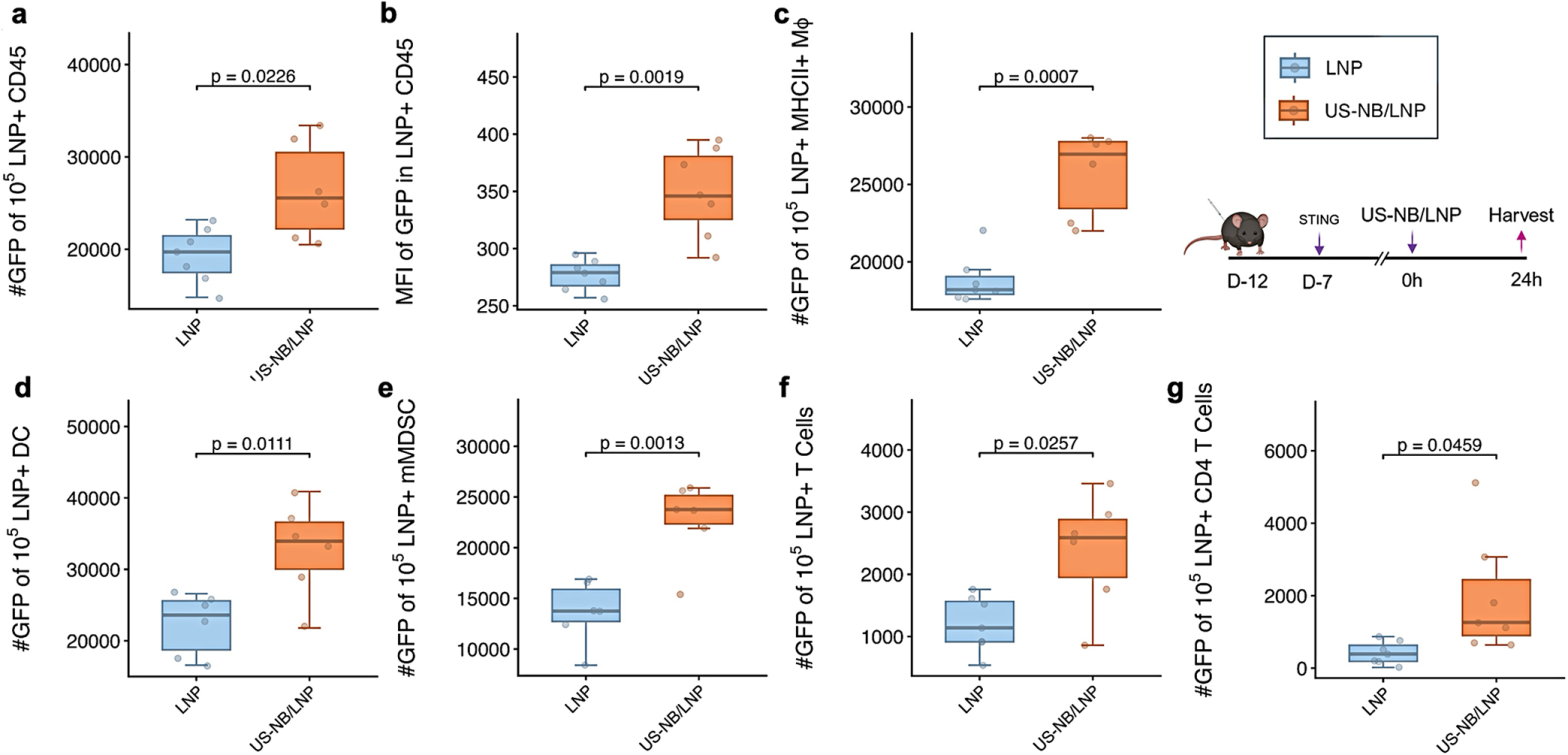
Improved transfection efficiency of US-NB/LNP in tumor-resident immune cells. Mice were inoculated with E0771.LMB cells and subcutaneously injected with 10 μg c-di-GMP 5 days postinoculation to enrich the T cell population, ensuring sufficient target immune cells at time of treatment. E0771.LMB tumors were treated when they were 40–60 mm^3^ with GFP mRNA-carrying fluorescent lipid nanoparticles. Tumors were harvested and assessed at a single cell level by flow cytometry. Within the population that was nanoparticle positive, (a) the proportion of immune cells (CD45^+^) expressing GFP and (b) the mean fluorescent intensity of that expression was compared. (c) MHCII^+^ macrophages, (d) dendritic cells, (e) mMDSC, (f) T cells and (g) CD4^+^ T cells were similarly quantified. Experiments were conducted with *n* = 6 mice per group. Comparisons were made between LNP (PBS + gene-carrying LNPs) and US-NB/LNP (nanobubbles + TUS + gene-carrying LNPs) groups. Statistics were performed using an unpaired student’s t test with Welch’s correction.

US-NB also increased the LNP-mediated transfection of T cells. Remarkably, upon nanobubble cavitation, double the number of LNP^+^ T cells expressed GFP (Figure 5f) compared to LNP delivery without US. Notably, the CD4 subset of T cells exhibited a 6-fold increase in transfection (Figure 5g). Typically, CD4^+^ T cells in the tumor are primarily regulatory or exhausted and therefore are a desirable target for various LNP-mediated therapeutic strategies.

Overall, these results show that US-NB can broadly boost LNP-mediated gene transfer and expression across both innate and adaptive immune compartments. By facilitating direct transfection of immune subsets central to tumor reprogramming, US-NB/LNPs may accelerate the development of antigen-specific antitumor responses, enhancing the overall efficacy of immunotherapies without the need for cell-specific targeting.

### Nanobubble Assisted Immunotherapy Significantly Improves Tumor Immunogenicity

We then investigated whether the combination of US-NB and LNPs (US-NB/LNP) could effectively reprogram the tumor immune microenvironment. Since effective antitumor responses rely on coordinated immune signaling, we assessed chemokines and cytokines that regulate recruitment and activation of innate and adaptive immune cells. For these studies, LNPs coencapsulated CTLA-4 and PD-1 siRNAs, a clinically efficacious immunotherapy. E0771.LMB tumors received three US-NB/LNP treatments at three-day intervals to match siRNA kinetics, enable repeated dosing, and capture delayed immune responses. Tumors and plasma were harvested 24 h after the final treatment from mice treated with US-NB/LNP, US-NB alone, LNP alone, or PBS, and analytes were quantified using a multiplex bead assay.

US-NB/LNP elevated chemokines associated with immune infiltration. The tumor and blood showed increased levels of CXCL10 (Figure 6a,b) and CCL2 (Figure 6c,d). For example, US-NB/LNP resulted in at least a ∼2-fold enhancement of CXCL10 in the tumor relative to the controls (Figure 6a). CXCL10 signaling recruits CD8^+^ T cells and NK cells into the tumor, generating a cytotoxic response. Similarly, US-NB/LNP generated a 2-fold increase in the CCL2 levels in the tumor compared to controls (Figure 6c). CCL2 is correlated with macrophage recruitment and neutrophil-mediated responses. Together, these changes indicate that US-NB/LNP establishes chemokine gradients that favor infiltration of effector and antigen-presenting cells.

**Figure 6:**
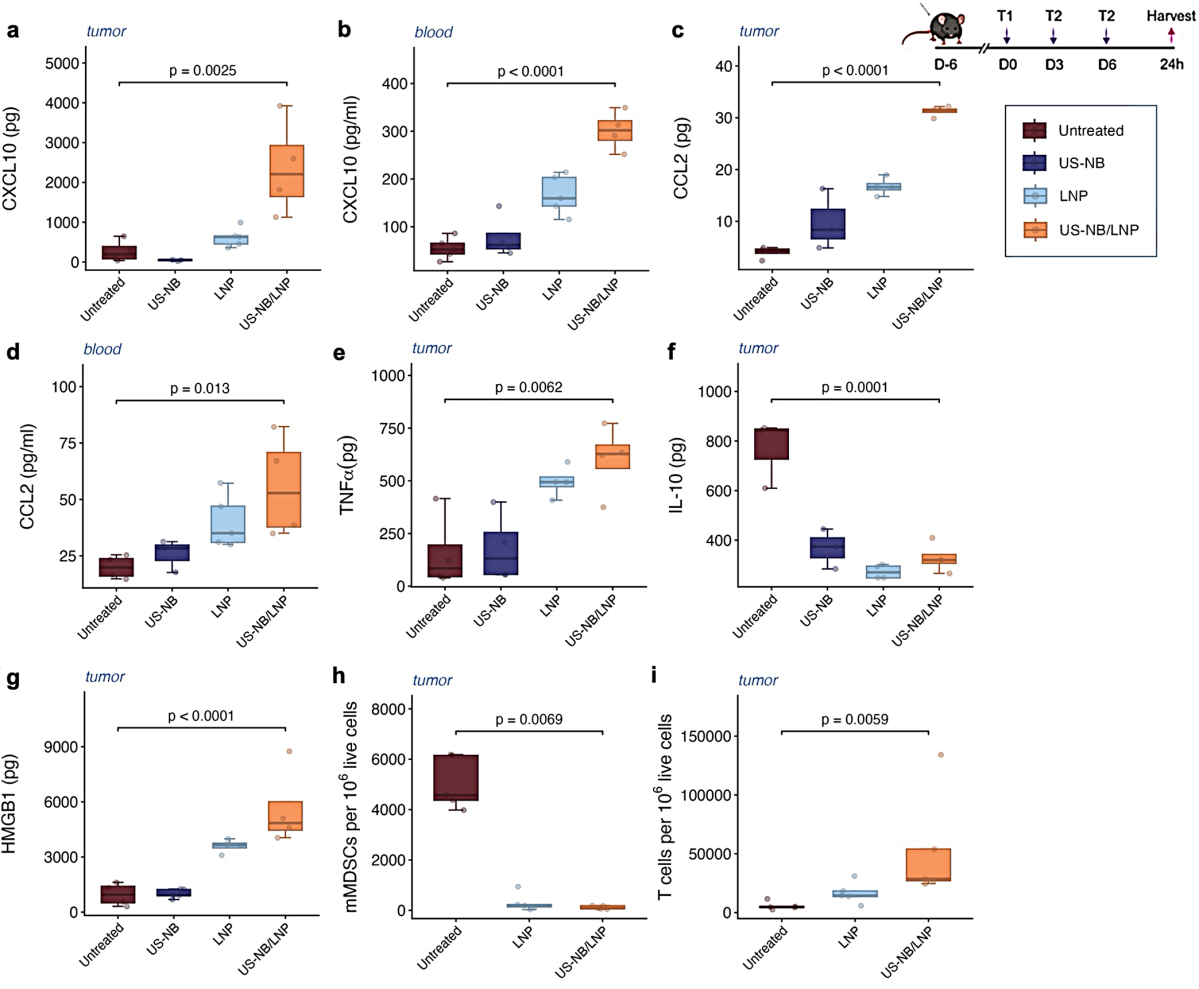
Immune signaling for combination LNPs to assess long-term changes. E0771.LMB tumors were treated on days 6, 9, and 12 and harvested 24 h after the third treatment. Signaling molecules were quantified using a multiplex bead assay (*n* = 4 mice per group). (a–d) Chemokines were measured in tumor homogenates and plasma for US-NB/LNP (Nanobubbles with therapeutic ultrasound, followed by gene-carrying lipid nanoparticles), LNP (PBS followed by gene-carrying lipid nanoparticles), US-NB (Nanobubbles with therapeutic ultrasound), and Untreated (PBS): (a) CXCL10 in tumor, (b) CXCL10 in blood, (c) CCL2 in tumor, and (d) CCL2 in blood. Similarly, (e) pro-inflammatory (TNFα) and (f) anti-inflammatory (IL-10) cytokines were measured across groups in the tumor. (g) HMGB1 in the tumor was quantified by an ELISA as a measure of ICD. (h, i) In a separate study using an identical schedule, E0771.LMB tumors were treated on days 6, 9, and 12 and harvested 24 h after the third treatment (*n* = 5 mice per group). Flow cytometry analysis was performed for (h) mMDSCs and (i) T cells. Statistics were carried out using a one-way ANOVA followed by Tukey’s post hoc test comparing the mean of each group to the others.

US-NB/LNP shifted the cytokine balance toward a pro-inflammatory, immunostimulatory profile. Within tumors, IFNγ and TNFα were upregulated by approximately 3.9-fold and 2.6-fold, respectively, relative to untreated controls (Figure S7 and 6e). In contrast, IL-10, a key immunosuppressive cytokine, was downregulated by about 2.3-fold in the US-NB/LNP group (Figure 6f). This coordinated increase in IFNγ and TNFα alongside a reduction in IL-10 is consistent with a transition from an immunosuppressive to an activated microenvironment that supports cytotoxic T cell and myeloid cell function. Intratumoral HMGB1 levels also increased by about 6.5-fold following US-NB/LNP treatment (Figure 6g), indicating enhanced release of this damage-associated molecular pattern, which promotes tumor immunogenicity and strengthens antigen presentation.

US-NB/LNPs enhanced the immunogenicity of the tumor. Tumors were evaluated at the same time point as the immune signaling studies using flow cytometry. Results showed mMDSCs, cells primarily responsible for immunosuppression in the tumor, had been depleted by more than 10-fold compared to the untreated controls (Figure 6h). In parallel, the increased CXCL10 expression is directly supported by the T cell enrichment of the tumor (Figure 6i). US-NB/LNPs led to a 6-fold increase in tumor-resident T cells, indicating successful immune-priming of the tumor.

In summary, these results show that US-NB/LNP therapy can transform the tumor microenvironment, promoting immune signaling pathways that favor infiltration and activation of cytotoxic and antigen-presenting cells and shifting the balance toward a pro-inflammatory, immunogenic state. The combined chemokine, cytokine, and cellular profile is characteristic of a microenvironment that can sustain robust antigen-specific responses and is associated with more durable therapeutic outcomes.

### Nanobubble-Assisted Immunotherapy Functionally Activates Immune Cells

Since US-NB/LNPs had already altered immune signaling and increased T cell infiltration, we next asked whether these changes translated into antigen-specific activation of myeloid and T cell populations. Tumors and tumor-draining lymph nodes (TDLN) were collected at the same 24 h time point as the cytokine and chemokine measurements and analyzed by flow cytometry for antigen-presenting myeloid cells and CD44^+^ activated T cells.

In the tumor microenvironment, US-NB/LNPs caused the expansion of functional antigen-presenting myeloid cells. Antigen-presenting macrophages increased by approximately 3.8-fold compared to LNP controls, and the population shifted toward a pro-inflammatory M1 phenotype, with M1 macrophages increasing more than 9-fold (Figure 7a,b). Dendritic cells showed a similar pattern of activation: antigen-presenting DCs rose by about 3.4-fold, and mature CD86^+^ DCs increased roughly 3.3-fold relative to LNP alone (Figure 7c,d). These data indicate that US-NB/LNPs not only expand intratumoral myeloid cells but also reprogram them toward an antigen-presenting phenotype.

**Figure 7:**
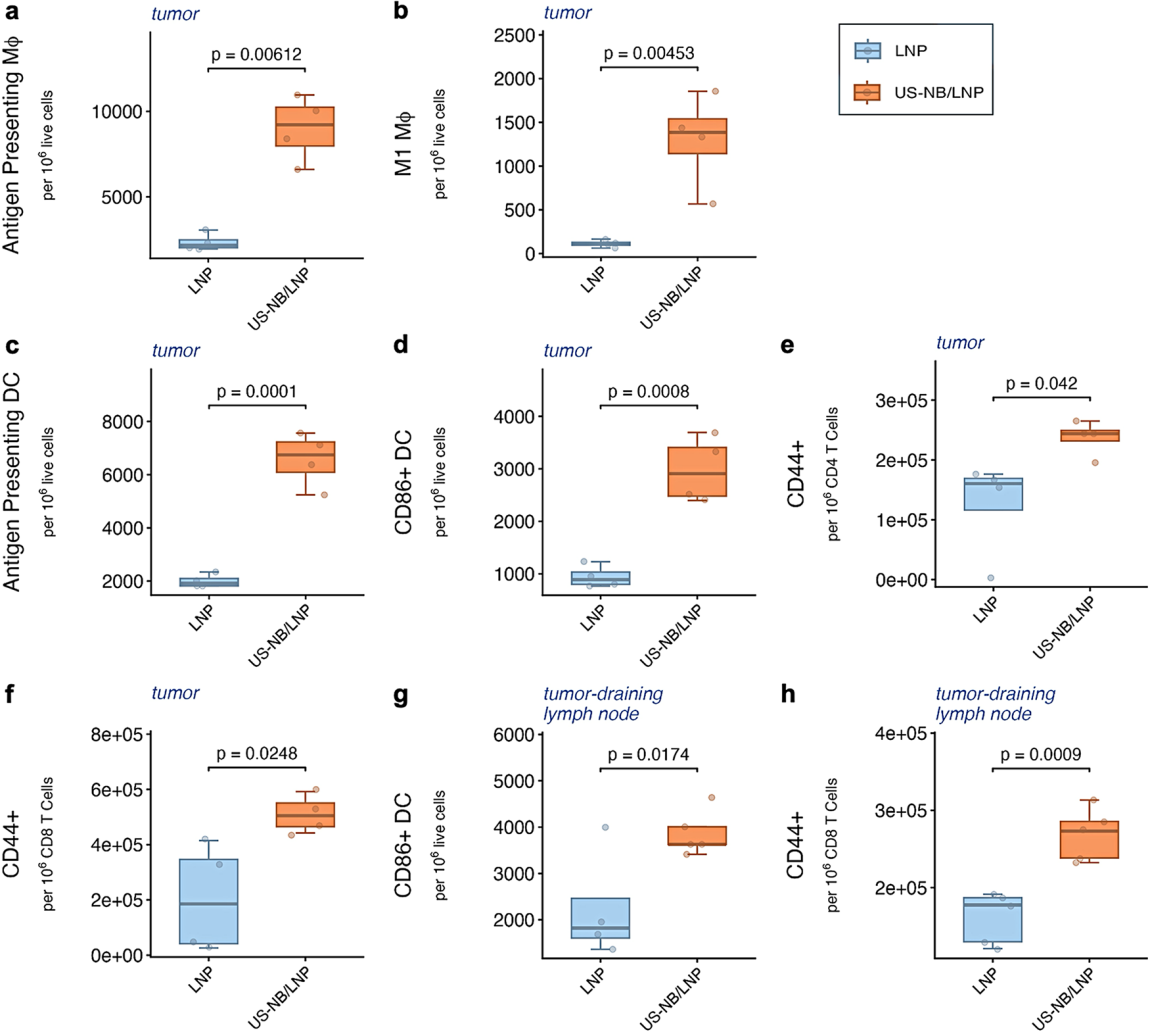
Activation of innate and adaptive immune populations. E0771.LMB tumor-bearing mice were treated on days 6, 9, and 12, and tissues were harvested 24 h after the final treatment (*n* = 4 biologically independent samples). Flow cytometry analysis was performed to quantify the activation of intratumoral myeloid populations, including (a) MHCII^+^ macrophages, (b) M1 macrophages, (c) MHCII^+^ dendritic cells, and (d) CD86^+^ macrophages. Adaptive immune activation was assessed via CD44 expression on (e) CD4^+^ and (f) CD8^+^ T cells within the tumor, as well as (g) CD86^+^ dendritic cells and (h) CD8^+^ T cells in the tumor-draining lymph nodes (TDLN). Comparisons were made between LNP (PBS + gene-carrying LNPs) and US-NB/LNP (nanobubbles + TUS + gene-carrying LNPs) groups. Statistics were performed using an unpaired student’s t test with Welch’s correction.

This myeloid engagement translated into enhanced T cell activation in the tumor. The frequency of activated CD4^+^CD44^+^ T cells increased by about 1.5-fold, while activated CD8^+^CD44^+^ cytotoxic T cells increased by approximately 2.8-fold compared to LNP-treated tumors (Figure 7e,f). In the context of the previously observed 6-fold increase in total intratumoral T cells, this enrichment of CD44^+^ subsets indicates that the infiltrating T cells are primarily antigen-experienced effector cells.

US-NB/LNPs enhanced systemic priming in the TDLN. Antigen-presenting DCs and mature CD86^+^ DCs in the dLN increased by roughly 2-fold relative to LNPs alone, consistent with activated DCs trafficking from the tumor to present antigen and provide costimulation (Figure 7g). In parallel, activated CD8^+^CD44^+^ T cells in the dLN expanded by about 1.5-fold (Figure 7h), supporting ongoing generation of new effector T cells that can reinforce the intratumoral response.

In summary, US-NB/LNPs converted mechanical remodeling and improved nanoparticle delivery into a coordinated, antigen-directed immune response. By expanding antigen-presenting macrophages and DCs by 3–9-fold in the tumor and increasing activated CD4^+^ and CD8^+^ T cells locally and in the draining lymph node by up to nearly 3-fold, the treatment results in both local myeloid reprogramming with sustained T cell priming. This establishes a tumor and lymph node environment that is functionally engaged in recognizing and responding to tumor antigens rather than maintaining an immunosuppressive state.

## Conclusions

In this study, we demonstrate that ultrasound-mediated nanobubble cavitation initiates a specific sequence of antitumor events. This process begins with physical ECM remodeling and leads to potent, antigen-specific immune activation. While previous strategies have focused on optimizing nanoparticle formulations, our results show that optimizing the tumor microenvironment itself is equally critical.^45^ Our data indicate that US-NB treatment reduced overall ECM stiffness to one-third of its baseline value and created a more uniform landscape. This physical normalization improved the volumetric distribution of lipid nanoparticles (LNPs) and significantly increased LNP uptake per immune cell. These changes led to a marked improvement in downstream transfection efficiency, particularly within difficult-to-transfect CD4^+^ T cells.^43^ This approach successfully reprogrammed the tumor microenvironment (TME) from an immunosuppressive, stroma-rich state to an inflamed, immunogenic phenotype.

While foundational studies have successfully established the efficacy of microbubble-mediated sonoporation for enhancing vascular permeability and drug accumulation,^46,47^ our work extends this paradigm to address the dense interstitial barrier. Elevated ECM stiffness appears to play a key role by supporting tumor growth via various mechanisms.^6^ The increased collagen deposition, ECM stiffness, and its heterogeneity support exponential growth partially due to biomechanical forces that strongly regulate the behavior of tumor cells and function of immune cells.^21^ Moreover, the stiff tumor ECM acts as a physical barrier impeding the infiltration of immune cells and effective drug delivery. To address this challenge, typical ultrasound interventions employ systemic administration of microbubbles, which have shown minimal tissue penetration resulting in the cavitation effects being primarily localized within the vascular and near-perivascular regions of tumors. While both nanobubbles and microbubbles share the advantage of having flexible, deformable shells, their size difference can lead to different biophysical behavior. Nanobubbles can distribute more efficiently within the dense and heterogeneous ECM of tumors. This enhanced tissue penetration and more homogeneous intratumoral distribution allow NBs to reach tumor regions that may be inaccessible to larger microbubbles, especially areas with high stromal density. Under ultrasound exposure, NBs can produce a mild cavitation response due to their lower gas content compared to microbubbles.^29^ This gentle acoustic impact reduces ECM stiffness (and interstitial pressure) without causing extensive cellular damage. By preserving the viability of immune cells, US-NB produces a more permissive niche for immunotherapeutic interventions. It should be noted that microbubbles could also achieve a relatively gentle impact when ultrasound parameters (e.g., frequency, pulse duration) are appropriately adjusted.^46^ However, the size and gentle acoustic impact of NBs provide a layer of precision and safety for US-NB-mediated tumor modulation without tissue ablation.^22^

In this study, we elected to utilize intratumoral injection to leverage NB’s nanoscale size, deformable shell, and compressible gas core to achieve complete tumor penetration. Nanobubble cavitation led to significant reductions in ECM stiffness, collagen cross-linking, and tissue heterogeneity, supporting increased drug delivery. This triggered release offers translational promise, as following ultrasound-mediated cavitation, the nanobubble phospholipid shell is metabolized through natural lipid degradation and cleared by the reticuloendothelial system, while the C_3_F_8_ gas core is eliminated via exhalation. Overall, therapeutic efficacy and favorable biocompatibility, low long-term toxicity, and efficient NB clearance has been shown in preclinical studies.^22,26,49^

US-NB and the reduced ECM stiffness allowed for improved delivery of LNPs. In the absence of US-NB, the intratumoral administration of LNPs resulted in limited distribution in the tumor, with the majority of the LNPs being localized in close proximity to their injection site.^35^ This is primarily a result of high interstitial pressures in tumors causing nanoparticles to rely on diffusive distribution.^51^ Considering the tumor periphery is a region of very high cell densities and pressures, it is not surprising the transport of the LNPs from the center (injection site) to the periphery of the tumor was limited. While larger in diameter than LNPs, nanobubbles exhibited superior tumor dispersion compared to LNPs because their highly compressible gas core and deformable phospholipid shell enables them to squeeze through the tumor dense matrix and irregular ECM pores, contrasting with the much less deformable, rigid structure of LNPs. In addition to US-NB’s impact on the broader distribution of LNPs in the tumor, the localized shock waves and microjets can sensitize cells to delivery.^52^ While therapeutic ultrasound-nanobubble cavitation generates strong mechanical forces that disrupt tumor tissue and enhance drug delivery, current evidence indicates that, when properly controlled, these forces do not facilitate tumor cell dissemination or increase metastasis risk, as cavitation-induced tissue disruption is localized, transient, and often accompanied by immune activation that can counteract metastatic spread.^53,54^ However, additional studies are needed to ensure that US-NB-induced cavitation does not contribute to metastatic spread. The sensitization of cells is typically attributed to the cavitation-induced activation of endocytic pathways.^47^ In addition to LNPs being able to disperse throughout the tumor, US-NB facilitates the internalization of LNPs by more immune cells as well as the internalization of a higher number of LNPs by each immune cell.

While this work focused on LNPs for immune-checkpoint silencing, US-NB can benefit many cancer therapies such as CAR-T cells, antibody treatments and cancer vaccines. Although LNPs are an increasingly popular choice for gene delivery, only a small portion of the gene cargo is intactly delivered to the cytosol. This is even more problematic in the case of LNP delivery to T cells. To direct nanoparticles to T cells, targeting schemes are employed by decorating the particles with targeting ligands,^55^ often antibodies. US-NB offers a convenient alternative by improving the transfection of pan-immune cells, exceptionally even T cells, without the need for additional modification of the nanoparticle. Thus, US-NB is agnostic to the type of LNP formulation or its cargo and can be a versatile companion for various applications of direct in vivo gene modification of antigen-presenting cells, T cells, or both innate and adaptive immune cells. In this study, we prioritized defining the mechanistic changes driving this improved delivery and the resulting functional activation of the tumor. We assessed this immune impact by characterizing specific shifts in immune signaling alongside immune cell activation and priming. These functional insights confirm the platform’s ability to convert physical remodeling into biological reprogramming, establishing the necessary foundation for future studies focused on long-term therapeutic outcomes.

In conclusion, nanobubble cavitation remodels the ECM, resulting in enhanced delivery and efficacy of immunotherapy. While most efforts focus on optimizing the delivery system to achieve improved delivery to tumors, US-NB seeks to optimize the tumor for delivery. Demonstrating strong efficacy in early stage breast cancer, US-NB therapy offers a flexible platform for targeting stroma-rich tumors through ECM remodeling and improved immunotherapy delivery. With further optimization of ultrasound parameters, this approach could be adapted for a wider variety of tumor sizes and cancer types, ranging from those with soft tissue characteristics, like melanoma, to highly complex structures such as pancreatic tumors.^10^ Overall, US-NB can become a drug-agnostic immunomodulatory intervention offering a wide applicability for patients with different treatment requirements.

## Methods

### Animal Ethics Statement

All animal experiments were performed under a protocol (protocol number: 2016–0115) approved by the Institutional Animal Care and Use Committee (IACUC) of Case Western Reserve University (CWRU).

### Animal Studies

E0771.LMB tumor cells (ATCC, Manassas, VA, USA) were cultured in DMEM supplemented with 10%FBS and 1%PS (Gibco, Evansville, IN, USA) at 37 °C in a humidified 5% CO_2_ incubator. 6–8 week C57BL/6J (Jackson Laboratories, Bar Harbor, ME, USA) were intradermally inoculated on the right flank with 250,000 tumor cells in 30 μL of DMEM. Mice were monitored daily for their weight and tumor volumes (length × width^2^ × 0.5), where length is the longest axis, and the width is its perpendicular. Mice were placed on a heating pad under 1–2% isoflurane for all treatments. Treatment area was initially shaved and chemically depilated whenever needed to prevent hair growth.

### Synthesis of Nanobubbles

NBs were formulated as previously described.^56^ Briefly, NBs were prepared by dissolving DBPC, DPPA, DPPE (Avanti, Birmingham, AL, USA), and mPEG-DSPE (Laysan, Arab, AL, USA) in propylene glycol (PG). A mixture of glycerol and PBS was then added to the lipid solution. Next, 1 mL of the lipid solution was aliquoted into a 3 mL vial, and the air inside was removed and replaced with C_3_F_8_ gas. To activate NBs, the vial was shaken using a VialMix shaker (Bristol-Myers Squibb Medical Imaging, Inc., N. Billerica, MA, USA) for 45s to induce bubble self-assembly. NBs were then isolated by differential centrifugation at 50 rcf for 5 min with the vial inverted. Nanobubbles were extracted from the bottom of the vial using a 21G syringe.

### Characterization of Nanobubbles

The size distribution and concentration of buoyant particles (bubbles) and nonbuoyant particles (lipid aggregations, micelles) were analyzed using resonant mass measurement (RMM) (Archimedes, Malvern Panalytical Inc., Westborough, MA, USA) with a calibrated nanosensor (100 nm – 2 μm). The sensors were precalibrated using NIST-traceable 565 nm polystyrene bead standards (ThermoFisher 4010S, Waltham, MA, USA).^20,57^ NBs were diluted 1:1000 in PBS, and at least 500 particles were measured per trial (*n* = 3). The average particle size (buoyant and nonbuoyant) and aggregation were further assessed using dynamic light scattering (DLS) (Litesizer 500, Anton Paar, Ashland, VA, USA). NBs were diluted 1:1000 in PBS and measured for intensity- and number-weighted size distributions. NBs underwent quality control by assessing their in vitro acoustic properties, including initial contrast enhancement and stability under ultrasound, using a tissue-mimicking agarose phantom.^20^ Nonlinear contrast images were continuously acquired using an ultrasound scanner (Vevo 2100, VisualSonics, New York, NY, USA) with nonlinear contrast-enhanced ultrasound at 18 MHz, 4% power, and 1 frame per second over 5 min. Raw data was recorded and analyzed using ImageJ software (freeware available from NIH). The region of interest (ROI) was defined, and total intensity was quantified using the Time Series Analyzer V3 plugin. From the data, initial signal enhancement, signal decay over time, and the percentage of remaining signal at 5 min were determined. The experiment was conducted in triplicate.

### Synthesis of Microbubbles

Microbubbles were formulated by dissolving DBPC, DPPA, DPPE (Avanti, Birmingham, AL, USA), and mPEG-DSPE (Laysan Bio, Arab, AL, USA) in propylene glycol (PG). A mixture of glycerol and PBS was then added to the lipid solution. Next, 1 mL of the lipid solution was aliquoted into a 3 mL vial, and the air inside was removed and replaced with C_3_F_8_ gas. To activate MBs, the vial was shaken using a VialMix shaker (Bristol-Myers Squibb Medical Imaging, Inc., N. Billerica, MA, USA) for 45 s to induce bubble self-assembly. Microbubbles were then isolated by multiple rounds of differential centrifugation in inverted vials. After the first spin at 50 rcf for 5 min, 500 μL was removed and the remainder of the solution was resuspended in 3 mL of PBS/PG/glycerol. The vial underwent a second centrifugation at 100g for 2 min and the liquid phase was removed. The remainder of the solution was resuspended in 2 mL of PBS/PG/glycerol.

### Therapeutic Ultrasound Treatment

NBs were intratumorally administered after a 1:10 dilution in sterile PBS, delivering a total of ∼7 × 10^7^ NBs in 20 μL into a tumor of ∼60 mm^3^. Treatments were delivered at the center of the tumor using a 29G1/2 insulin syringe (Exel International, Quebec J6T 0E3, Canada). Therapeutic ultrasound (TUS) was applied on the tumor using an unfocused transducer with a 1 cm^2^ effective radiating area (Sonicator 740, Mettler, Anaheim, CA, USA) at 3.3 MHz, 2.2 W, and a 50% duty cycle for 1 min. Ultrasound coupling gel was applied at a height of 1 cm, measured from the probe to the flank tumor. TUS parameters included a pulse repetition frequency (PRF) of 100 Hz, a pulse length of 10 ms, and an estimated peak negative pressure (PNP) amplitude of 0.25 MPa. Treatment groups included: US-NB (Nanobubbles with therapeutic ultrasound), US-NB/LNP (Nanobubbles with therapeutic ultrasound, followed by gene-carrying lipid nanoparticles), US/LNP (PBS with therapeutic ultrasound, followed by gene-carrying lipid nanoparticles), US (PBS with therapeutic ultrasound), LNP (PBS followed by gene-carrying lipid nanoparticles), Untreated (PBS alone). All treatments had the same number of injections and total volume, supplemented with PBS when needed. All LNP treatments were conducted immediately after therapeutic ultrasound, at the same injection site as the initial NB injection.

### Treatment Schedules

Treatments were typically conducted at tumor volumes of 40–60 mm^3^, 12 days after inoculation. The mRNA transfection study (Figure 5) included a subcutaneous pretreatment on day 5 with 10 μg c-di-GMP (STING agonist, InVivoGen, San Diego, CA) to enrich the T cell population. Immunogenicity studies (Figure 6 and 7) were conducted 24 h after 3 treatments, 3 days apart to assess long-term antigen-initiated responses.

### Volumetric Nonlinear Contrast-Enhanced Ultrasound Imaging

Three-dimensional tumor imaging was performed using a preclinical ultrasound imaging system (Vevo F2, FUJIFILM VisualSonics, NY, USA) equipped with a UHF29X transducer (15–29 MHz bandwidth). Imaging was conducted in dual B-mode and 3D nonlinear contrast (NLC) mode, with an NLC transmit frequency of 17.5 MHz (10% power, 0 dB contrast gain, 0 dB gain, 50 dB dynamic range, 5 frames per second, 0.2 mm step size). The focus was aligned to the center of the tumor and imaging began immediately after NB injection. NLC signal of the ROI was analyzed using an uncompressed, linear scale and quantified using MATLAB (MathWorks, Natick, MA, USA). To evaluate NB spread, % area containing signal above baseline was measured per ROI and averaged across frames. Nanobubble cavitation was evaluated by quantifying total NLC signal intensity, pre- and post- therapeutic ultrasound and then averaged across frames.

### In Vivo US-Shear Wave Elastography (SWE)

Tumor elasticity was measured using Siemens Acuson S2000, (Siemens Healthineers, Munich, Germany) equipped with a 9L4 linear array probe (4–9 MHz bandwidth). Tumor bearing mice were placed under 1–2% isoflurane on a heating pad under a fixed position for imaging. To ensure a consistent scanning setup, a depth of 6.5 cm was maintained using a standoff gel pad (Aquaflex, Santa Ana, CA, USA) and a uniform layer of acoustic coupling gel. Scans were performed perpendicular to the tumor following manufacturer-recommended settings for shear wave elastography (SWE). Mice were imaged at tumor volumes of approximately 50 mm^3^, and SWE measurements were taken immediately postultrasound treatment and then daily through day 5. Representative B-mode images defined region-of-interest (ROI) grids encompassing the entire tumor slice. Within each tumor ROI, elastic moduli were determined from three independent sampling points at distinct locations. At each sampling point, the ultrasound system measured shear wave velocity and automatically calculated Young’s modulus based on the relationship between tissue density and shear wave velocity. The reported elastic modulus for each tumor was the average of measurements from all three sampling points. Measurements were concluded when tumor growth exceeded ROI grid dimensions, ensuring consistent measurement geometry throughout the study. Daily caliper measurements confirmed that tumor volume remained relatively stable throughout the measurement period, allowing isolation of the effects of ECM remodeling independent of tumor growth. US-NB (Nanobubbles with therapeutic ultrasound) and Untreated (PBS) were compared every 24 h, with the exception of an additional measurement immediately after TUS for the US-NB group.

### In Vitro Phantom Validation for US-Shear Wave Elastography (SWE)

To validate SWE measurements, polyacrylamide phantoms were made to have specific moduli of 2- and 40 kPa. The preparation process followed a previously reported method.^58^ In summary, phantom solutions were prepared using 40% acrylamide at concentrations ranging from 6.4 to 26.5 wt % (Supporting Table 1). Cross-linking was initiated through free radical polymerization, facilitated by TEMED and APS, with polymerization occurring at room temperature within designated containers. Each phantom contained 0.095 wt % bis-acrylamide as a cross-linking agent. After polymerization, the phantoms were stored in PBS to maintain stability.

### Histological Analysis

Tumors were harvested and washed in PBS. Samples were placed in 30% sucrose for 48 h followed by 4% PFA for another 24 h at 4 °C. Tumors were then washed and placed in OCT for 72 h at −80 °C before being cryo-sliced at 12 μm. Cell death IF staining used an Alexa Fluor 488 polyclonal Ab specific for cleaved caspase 3 (Life Technologies, CA, USA) and DAPI mounting media (Abcam, Waltham, MA, USA). Spatial evaluation of LNP spread was similarly conducted using DiR tagged LNPs and DAPI mounting media. H&E and picrosirius staining for collagen were conducted by the CWRU Tissue Core. Cell death IF, LNP spread and H&E slides were imaged on the Zeiss Axio Z1 (Carl Zeiss Microscopy, White Plains, NY, USA). Picro Sirius Red Collagen staining was imaged with the Zeiss Axio Z1scanner. All images were analyzed via MATLAB.

### Synthesis of Lipid Nanoparticles

Lipid nanoparticles (LNPs) loaded with siRNA were synthesized as previously described.^59^ DLin-MC3 (Cayman Chemical), cholesterol (ovine) (Avanti Lipids), Distearoylphosphatidylcholine (DSPC) (Avanti Lipids), 1,2- dimyristoyl-rac-glycero-3- methoxypolyethylene glycol- 2000 (DMG- PEG2000) (Avanti Lipids) and 1,2- Distearoyl-*sn*-glycero- 3- phosphoethanolamine-mPEG (DSPE-mPEG2000) (Laysan Bio) were mixed in a solution of chloroform at a ratio of 50:38:10.5:1.4:0.1. Lipid solutions were evaporated at room temperature and the resulting films were hydrated with siRNA in nuclease-free acetated buffer in a 1:3 ratio (v/v) and mixed rapidly. The lipid-drug solution was then probe sonicated at 20% PW at 30s on, 10s off for 5 min. The solution was encapsulated in 300 kDa membranes and dialyzed against 1× nuclear free-PBS for 16–18 h. LNPs loaded with mRNA were made with an identical lipid solution but with the ionizable cationic lipid switched from D-Lin-MC3 to SM102 of the same molar ratio. EGFP mRNA (TriLink Biotechnologies) was reconstituted in nuclease- free citrate buffer and the lipid solution was mixed with the mRNA at a 1:3 ratio (v/v) through syringe mixing. The solution was encapsulated in 20 kDa membranes and dialyzed against 1× nuclear free-PBS for 16–18 h. The resulting nanoparticles were concentrated by centrifugation at 3000 rpm in an Amicon filter unit and used within 3 h of formulation. When fluorescently tagged, 0.2–0.5% DiR′ was added to the lipid solution, before film formation.

### Characterization of Lipid nanoparticles

LNPs were characterized for their hydrodynamic size and surface charge using dynamic light scattering (DLS) and ζ-potential (Anton Parr, Ashland, VA, USA). Samples were diluted in 1:1000 PBS and results were averaged across triplicates of three independent measurements. Drug loading was determined using Quant-it Ribogreen tests (Thermofisher, Waltham, MA, USA) after lysis with Triton-X 100. Total RNA of lysed and unlysed nanoparticles was quantified to determine both the encapsulation efficiency and input loading. In vivo treatment volumes were then finalized by drug content.

### Flow Cytometry

Harvested tumors were cut into smaller fragments using surgical scissors and then digested in Liberase (0.4 mg/mL, Roche, IN, USA) and DNase (0.2 mg/mL, Roche) in RPMI-1640 for 20 min at 37 °C. Digested tumors and tumor-draining lymph nodes were then mechanically dissociated and processed through 70 μm nylon mesh strainers twice. These single cell suspensions were first incubated with CD16/CD32 Fc block (Biolegend, San Diego, CA, USA) followed by immunophenotyping with fluorescent antibody (Supporting Table 2) staining. For viability assessment, cells were counterstained with DAPI or Zombie UV (BD Biosciences, Franklin Lakes, NJ, USA). All cells were read with the BD-LSR Fortessa and analyzed using FlowJo. The representative gating strategy is shown in Figure S6.

### Measurement of Cytokine, Chemokine and DAMPs

Blood was collected retro-orbitally in heparin tubes and plasma was collected through centrifugation and stored at −80 °C. Tumors were submerged in 5 mL PBS and homogenized with a tissue homogenizer and then stored in −80 °C. Samples underwent 3 freeze–thaw cycles and were centrifuged before the final aliquot storage in −80 °C. Multiplex immunoassay (LegendPlex, Biolegend) was used to assess CCL2, CXCL10, IL-10, IFNγ and TNFα following the manufacturer’s instructions and measurements were quantified using BD-LSR Fortessa. HMGB1 in tumor tissues was quantified with an ELISA (CusaBio, Houston, TX, USA) following the manufacturer’s instructions and absorbance readings were measured using a plate reader (Biotek, Winooski, VT, USA).

### Statistics

Statistical analyses were conducted using R, GraphPad Prism and MATLAB, and data is represented as mean ± SD. Unpaired *t* tests and one-way analysis of variance (ANOVA) were performed with the appropriate post hoc tests. Multiple comparisons were conducted by comparing each group to each other and *p* < 0.05 was considered significant.

## Supplementary Material



## Notes

Formulation details of polyacrylamide phantoms, flow phenotyping for immune cell populations, characterization of nanobubbles and microbubbles, SWE phantom validation, SWE qualitative measurements and tumor growth, histological evaluation of extracellular remodeling in the tumor microenvironment, characterization of lipid nanoparticles, representative flow gating strategy, immune signaling (IFNγ) (PDF)
