# Supplementary material for: Enhanced Delivery of Lipid Nanoparticle-Based Immunotherapy by Modulating the Tumor Tissue Stiffness Using Ultrasound-Activated Nanobubbles
Source: ACS Nano. 2026 Jan 28;20(5):4592–606. doi: 10.1021/acsnano.5c21787 (PMC12885110; doi:10.1021/acsnano.5c21787)
Supplement: Supplementary file 1 [file nn5c21787_si_001.pdf]

SUPPORTING INFORMATION

Enhanced delivery of lipid nanoparticle-based immunotherapy by modulating the tumor tissue stiffness using ultrasound-activated nanobubbles

Anubhuti Bhalotia[1], Diarmuid W. Hutchinson[1], Theresa Kosmides[1], Pinunta Nittayacharn[2,3], Meghna Mehta[1], Arya Iyer[1], Andrew Cheplyansky[1], Koki H. Takizawa[1], Abraham Nidhiry[1], Anna M. Dever[1], Kyle A. Cousens[1], Inga M. Hwang[1], Gopalakrishnan Ramamurthy[3], Agata A. Exner[1,3,4]*, Efstathios Karathanasis[1,4]*

[1] Department of Biomedical Engineering, School of Medicine, Case Western Reserve University, Cleveland, Ohio 44106, USA

[2] Department of Biomedical Engineering, Faculty of Engineering, Mahidol University, Phuttamonton, Nakorn Pathom, 73170, Thailand

[3] Department of Radiology, School of Medicine, Case Western Reserve University, Cleveland, Ohio 44106, USA

[4] Case Comprehensive Cancer Center, School of Medicine, Case Western Reserve University, Cleveland, Ohio 44106, USA

* Co-corresponding authors: Efstathios Karathanasis (stathis@case.edu) and Agata Exner (agata.exner@case.edu)

Contents

Table 1. Formulation details of polyacrylamide phantoms
Table 2. Flow phenotyping for immune cell populations
Figure S1. Characterization of nanobubbles and microbubbles
Figure S2. SWE phantom validation
Figure S3. SWE qualitative measurements and tumor growth
Figure S4. Histological evaluation of extracellular remodeling in the tumor microenvironment
Figure S5. Characterization of lipid nanoparticles
Figure S6. Representative flow gating strategy
Figure S7. Immune signaling (IFNγ)

Theoretical elastic modulus	Acrylamide (mL, wt.%)	Bis-acrylamide (mL, wt.%)	Solvent (mL)	APS (mL)	TEMED (μL)
**1 kPa**	(1.7, 6.4)	(0.5, 0.095)	7.8	0.5	50
**10 kPa**	(4.0, 15.2)	(0.5, 0.095)	5.5	0.5	50
**18 kPa**	(7.0, 26.5)	(0.5, 0.095)	2.5	0.5	50

Table 1. Formulation details of polyacrylamide phantoms (10 mL batch)

Cell population	Immunophenotype
Pan-immune cells	CD45+
Dendritic cells	CD11b+/F4-80-/CD11c+
Macrophages	CD11b+/F4-80+
mMDSCs	CD11b+/F4-80-/Ly6chiLy6G-
T cells	CD3e+/CD4+ or CD8+
T cell activation	T cells/CD44+
Antigen-presenting cell	Myeloid cells/MHC2+
M1 macrophages	CD11b+/F4-80+/MHC2+CD86+

Table 2. Flow phenotyping for immune cell populations

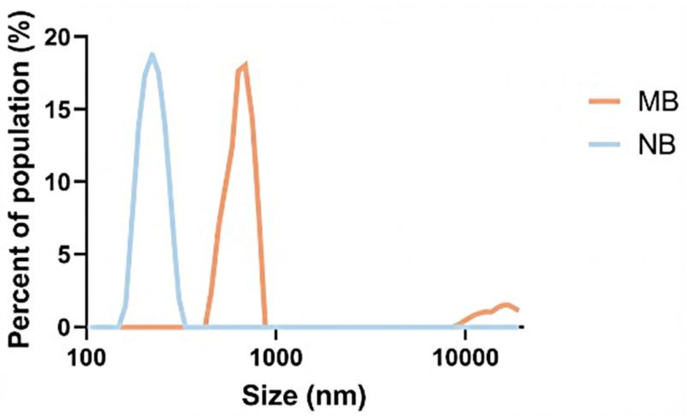

Figure S1. Characterization of the hydrodynamic diameter of nanobubbles and microbubbles in PBS.

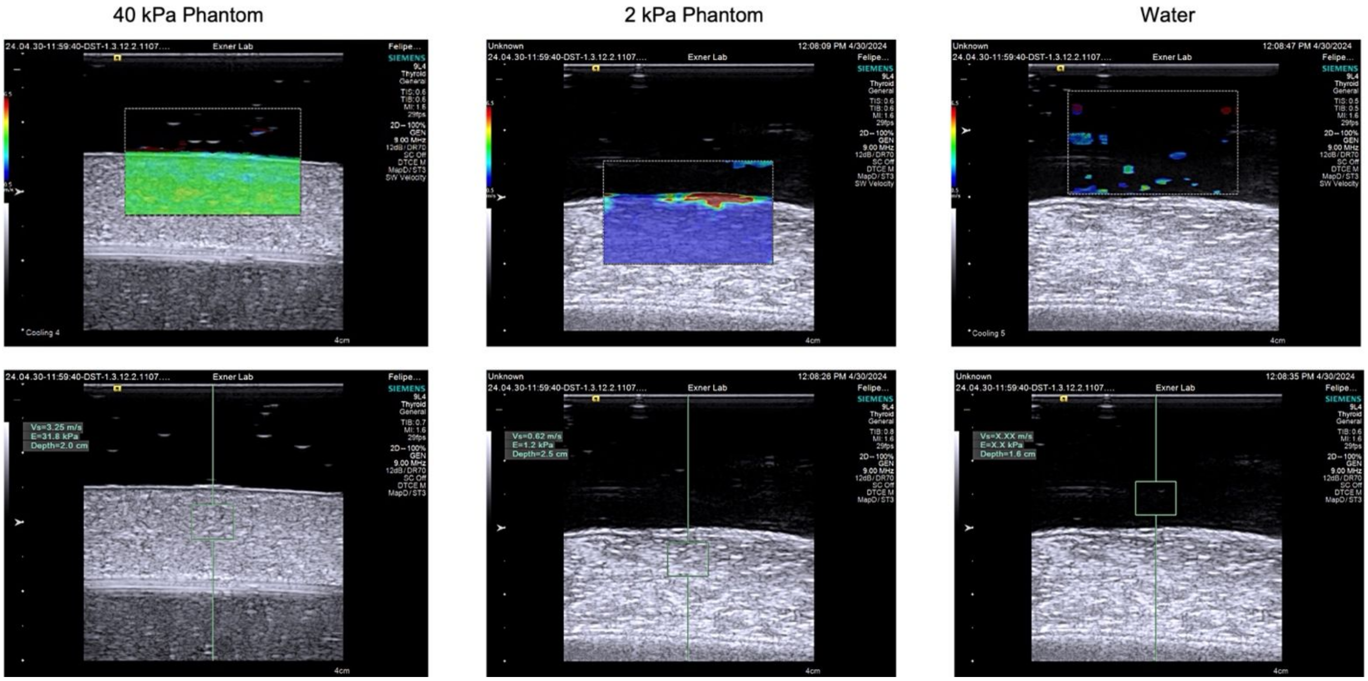

Figure S2. SWE phantom validation. Polyacrylamide phantoms were made based on the measurements in Table 1. Phantoms were placed in water and imaged using the Siemens 2000 using the 9L4 probe. Virtual Touch Imaging and Quantification Mode was used. The top row shows the qualitative elasticity map, while the bottom row displays the quantitative elasticity values measured within the region of interest (small box)

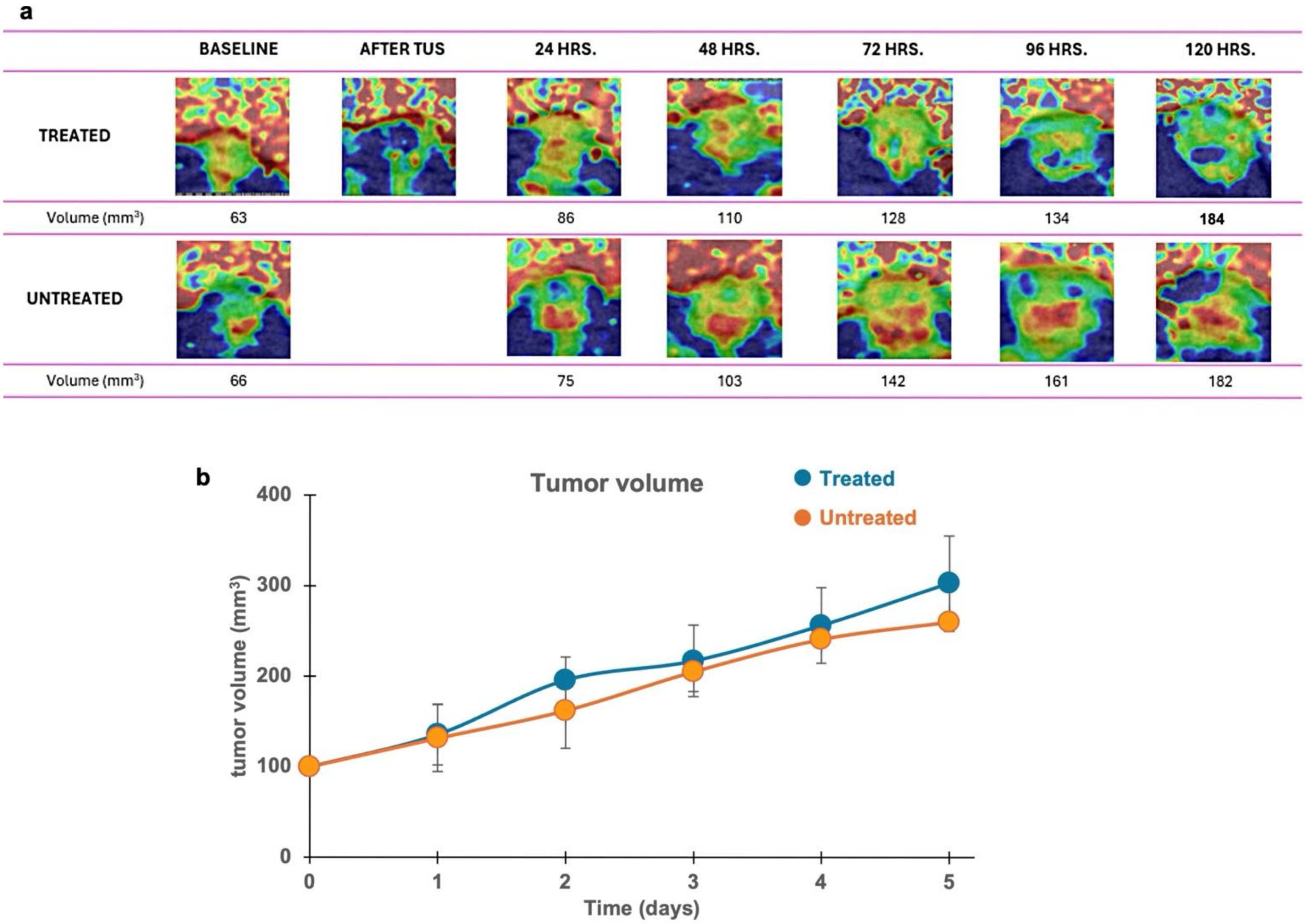

Figure S3. SWE qualitative measurements and tumor growth. (a) Uncropped elastograms for qualitative measurements. Due to the high spatial stiffness of the standoff gel pads used for the SWE setup, the elastograms were cropped to the tumor ROI and kept to their original size for ease of viewing of the growing tumor alone. Measurements taken by the Siemens 2000 using the 9L4 probe. Measurements are taken daily for 5 days. Set up was kept constant by regulating depth, gel thickness and probe angle. Qualitative elastograms are reported with the range of soft (blue) to hard tissue (red). **(b)** Tumor growth curves for the US-NB treated and the untreated groups.

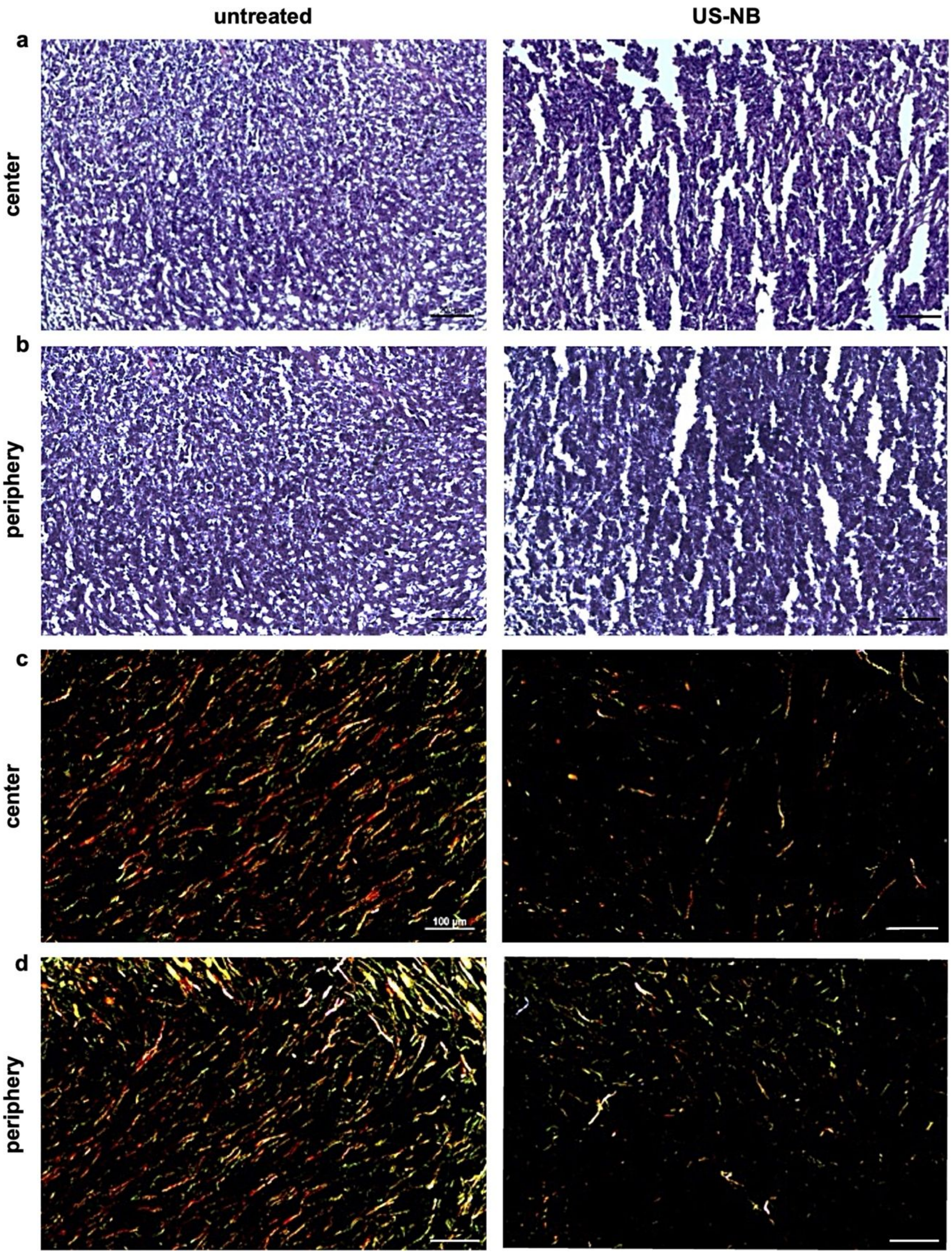

Figure S4. Histological evaluation of extracellular remodeling in the tumor microenvironment. E0771.LMB tumors were treated at 40-60 mm^3 and harvested 24 hours post-treatment for histological analysis (n=5 mice per group). Representative images with H&E staining show **(a)** the center and **(b)** the periphery of the tumor with or without US-NB. Representative images with Picrosirius Red staining visualized under polarized light show collagen content in **(a)** the center and **(b)** the periphery of the tumor with or without US-NB.

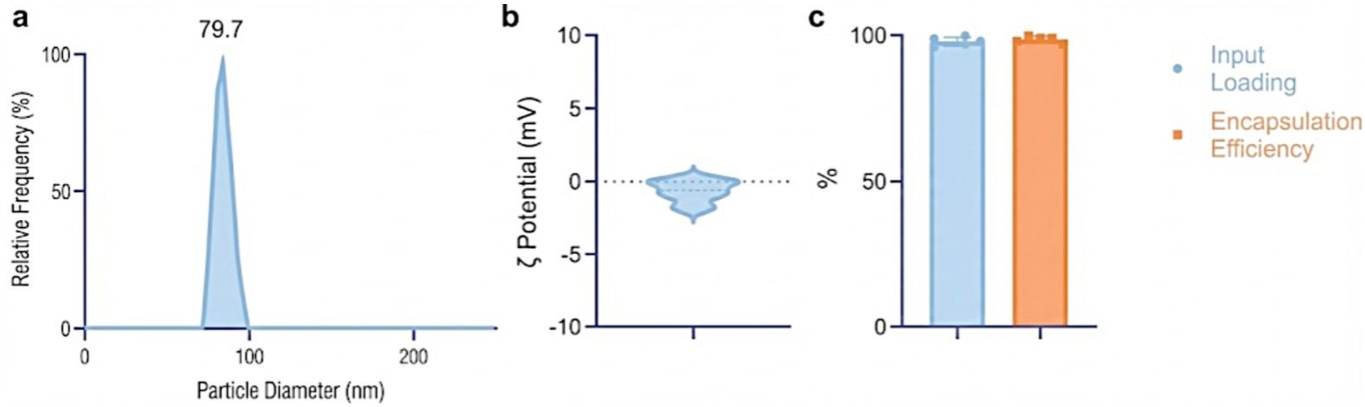

Figure S5. Characterization of lipid nanoparticles. Lipid nanoparticles carrying were formulated with the sonication method and characterized after 16-18 hours of dialysis. (a) Dynamic light scattering measurements were taken in PBS. The number-weighted distribution is visualized through a histogram to assess its dispersion. (b) Zeta potential measurements for surface charge evaluation are taken in DI water at RT averaged across a sample size of 3. (c) Drug input loading and encapsulation efficiency are taken by a fluorescent plate reader assay. Particles are lysed with 0.1% Triton X-100 at 37°C. Input loading is calculated considering the initial mass of drug added during formulation. Encapsulation efficiency is calculated by comparing lysed and un-lysed particles to determine the % of siRNA on the surface. N=3 was used for assessment.

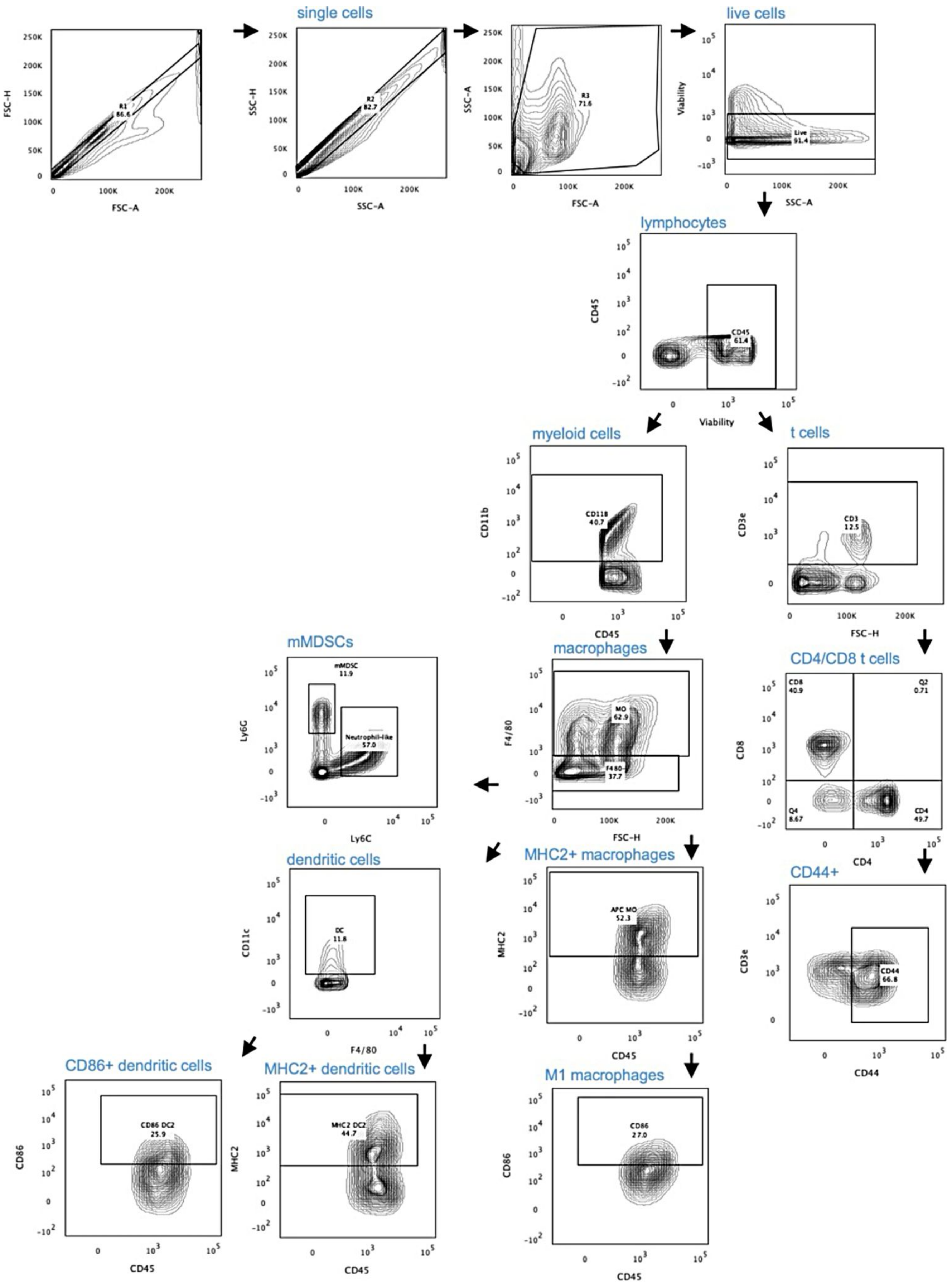

Figure S6. Representative flow gating strategy.

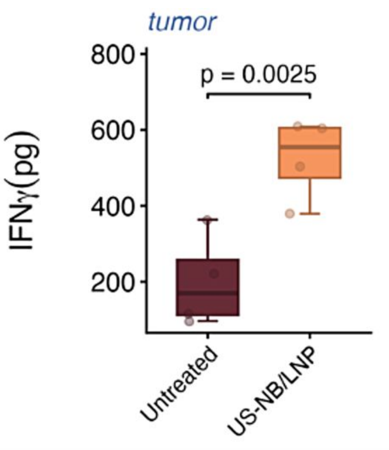

Figure S7. Immune signaling for the US-NB/LNP treatment to assess long-term changes (IFNγ). E0771.LMB tumors were treated on day 6,9,12 and harvested 24 hours after the third treatment. IFNγ was assessed for the NB+TUS/LNP and PBS groups using a multiplex bead assay. All concentrations were multiplied by the homogenization volume. All injection volumes across groups were kept constant. Statistics were carried out using a One-way ANOVA comparing the mean of each group to the others**.**
